# Self-reported dental hygiene, obesity, and systemic inflammation in a pediatric rural community cohort

**DOI:** 10.1186/1472-6831-10-21

**Published:** 2010-09-18

**Authors:** Stephanie J Frisbee, Christopher B Chambers, Jefferson C Frisbee, Adam G Goodwill, Richard J Crout

**Affiliations:** 1 Department of Community Medicine, School of Medicine, West Virginia University, Morgantown, West Virginia, USA; 2 Department of Dental Practice and Rural Health, School of Dentistry, West Virginia University, Morgantown, West Virginia, USA; 3 Center for Cardiovascular and Respiratory Sciences, School of Medicine, West Virginia University, Morgantown, West Virginia, USA; 4 School of Dentistry, West Virginia University, Morgantown, West Virginia, USA; 5 Department of Physiology and Pharmacology, School of Medicine, West Virginia University, Morgantown, West Virginia, USA

## Abstract

**Background:**

A growing body of epidemiologic evidence links oral health, obesity, and cardiovascular health, though few studies have reported on these relationships in children. While underlying mechanisms are unclear, adult studies have suggested sub-acute systemic inflammation, also implicated in the etiology of both obesity and cardiovascular disease. This study investigated associations between self-reported dental hygiene, obesity, and systemic inflammation in children.

**Methods:**

128 children < 19 years of age from rural counties in West Virginia participated in a community-based health screening that included anthropometric assessments, blood collection, and a questionnaire about dental hygiene and self-assessed oral health.

**Results:**

Participants ranged from 3.0-18.7 years. Univariate analysis demonstrated an association between parent-reported dental hygiene, including frequency of preventive dental care and parent-assessed overall dental health, and markers of systemic inflammation but not obesity. In multivariable regression, parent-assessed overall dental health and obesity were independent predictors of systemic inflammation, after adjustment for age, gender, and parent education.

**Conclusions:**

This is the first known study of the association between dental hygiene, obesity, and systemic inflammation in children. These results highlight the importance of preventive dental care in overall, systemic health in children and are consistent with previous reports in adults.

## Background

There have been substantial recent research efforts investigating the relationship between oral and systemic health, aided in part by the focus given the topic by the 2000 Surgeon General's report on "Oral Health in America" [1]. This report cited that dental caries affects almost ⅔ of children ages 5-17, with the burden of disease higher in poorer children [1]. Substantial socioeconomic and geographic oral health disparities have also been noted. Untreated tooth decay has been reported in three times as many children aged 6-11 from families with incomes below the U.S. federal poverty line compared to children from families with incomes above the poverty line (12% vs. 4%, respectively) [2]. Geographic disparities in adult oral health are particularly seen in areas of Appalachia, especially West Virginia, Kentucky, Louisiana, and Arkansas [3]. In children in West Virginia, 65.6% of children had at least 1 cavity by age 8, a proportion well above the national average [4].

In adults, there is a growing literature linking oral and periodontal health to increased risk for chronic health conditions, including obesity and cardiovascular disease. Some studies have reported a link between body mass index (BMI), as a proxy measure for obesity, and periodontal pockets [5]. Additional epidemiologic studies have associated poor oral health with increased risk for myocardial infarction and coronary atherosclerosis [6-14]. Systemic inflammation and/or bacterial pathogenesis have been identified as possible mechanistic pathways. Investigators have reported a relationship between the cumulative burden of periodontal pathogenic burden and coronary heart disease [15], the presence of periodontal bacteria in atherosclerotic plaques [16-19], and elevated levels of systemic C-reactive protein (CRP) and interleukin(IL)-6 [20]. Further, recent reports of randomized trials have reported improvement in systemic inflammation and endothelial function after treatment for periodontitis [21-26].

Currently, the body of scientific knowledge from studies of associations between oral health and obesity or cardiovascular health in children is not as well developed as it is in adults. To date, many studies on oral health in children have focused on issues related to access and efficacy of treatment recommendations [27-32]. Recent studies have reported a link between childhood oral health and obesity [33-36], though the observation of a positive correlation between dental caries and obesity has not been universal [37,38] leading to the recommendation of additional study [39]. Further, understanding an etiologic (causative) compared to correlative association between dental caries or other indicators of dental health and obesity or cardiovascular disease risk factors in children will likely require consideration of complex interrelations between nutritional status and habits, socioeconomic status, general health habits, and family influences among other factors [40]. However, given the demonstrated association between oral health, obesity, and cardiovascular disease in adults, and that a plausible biologic mechanism is cumulative pathogenic burden leading to systemic inflammation, early emphasis on good oral health in children would be prudent. In particular, routine preventive care and robust dental hygiene, given the well established link between dental hygiene habits and oral health, would be highlighted to forestall or avoid adverse pathogenic effects.

The purpose of this exploratory study was to assess the association between self- and parent-reported dental hygiene and self- and parent-assessed oral health, obesity, and systemic inflammation in children living in rural communities. An association between dental hygiene and self- or parent-assessed oral health and systemic inflammation after controlling for measures of obesity would provide preliminary support for an etiologic link between these conditions in children.

## Methods

### Participants

Participants were recruited from 5 different counties in West Virginia. Counties ranged in rurality from 3-9 on the United States Department of Agriculture Economic Research Service 2003 Rural-Urban Continuum Codes (9 being the most rural). Children and families in these counties were invited to participate in a comprehensive health screening taking place in their community during the spring-fall months of 2006. As there were no exclusion criteria for participation in the health screenings, participants in this study are considered a cross-sectional, convenience sample. In total, 128 children < 19 years of age participated in the health screenings. The average age of these participants was 11.1 ± 2.9 (standard deviation) years (range: 3.0-18.7) and 52% were girls. All methods and protocols were approved by the West Virginia University Institutional Review Board, and participants completed the appropriate informed consent procedures.

### Data Collection

Mobile data collection teams were stationed in community-based facilities for morning health screenings (7 AM-11 AM). Participants, having completed ≥8 hour fast, underwent standard anthropometric assessments that included height, weight, hip and waist circumference, and blood pressure (Omron HEM-711AC, Omron Healthcare, Kyoto, Japan). All anthropometric measures were taken in duplicate and results were averaged for analysis. For BMI, age- and gender-based percentiles were calculated using the Epi Info™ Nutrition module (Version 3.3.2, Centers for Disease Control and Prevention (CDC), Atlanta, Georgia, USA).

Participants provided a blood sample for determination of a fasting lipid profile and systemic inflammation; blood glucose levels were determined immediately (FreeStyle Flash Blood Glucose Monitoring System, Abbott Laboratories, Abbott Park, Illinois, USA). Children ≥10 years of age completed questions about health and lifestyle habits including a questionnaire about their dental health and dental hygiene practices, for which questions were derived in part from the National Health and Examination Survey (NHANES) developed as part of a multi-site study of dental health in Appalachia [41]. Parents of all children completed a similar questionnaire about their child's dental health and dental hygiene practices, similarly developed [41].

### Biochemical Analysis

All physiologic samples were processed at the time of screening, with plasma fractions snap-frozen on dry ice. Plasma samples were analyzed in a nearby hospital laboratory to obtain a fasting lipid profile (total cholesterol, low density lipoprotein (LDL) cholesterol, high density lipoprotein (HDL) cholesterol, triglycerides). Endocrine, cytokine, and other inflammatory markers, including insulin, were obtained from frozen plasma using the Luminex100^® ^system with the appropriate Lincoplex^® ^multiplex assay kits and protocols from LincoResearch (Luminex Corporation, Austin TX; Linco Research Coproration now Millipore Corporation, Billerica, Massachusetts, USA). Concentrations for all markers determined via the Luminex100^® ^system were obtained in duplicate; only concentrations with a coefficient of variation ≤ 0.5 were included. As sharply elevated CRP is considered indicative of acute infection, individuals with CRP > 10 mg/dL were excluded from analysis of variance (ANOVA) and regression statistical analysis [42]. Of the 128 participants in this study, 91 children contributed blood samples. Of these, two samples were excluded due to concerns about fasting or limited volume, seven excluded due to indication of acute infection, and nine excluded per data quality control procedures, resulting in a final sample of n = 73 available for statistical analysis.

### Statistical Analysis

All statistical analyses were performed with SPSS (SPSS Inc., Chicago, Illinois, USA). To better approximate normality in distribution, BMI z-score and not BMI was used in statistical analyses. Univariate analyses (ANOVA) were performed to identify associations to pursue with multivariable analysis. As univariate analysis was being used to guide further analysis and not to explicitly test hypotheses, correction for multiple testing was not used. For all multivariable models using ordinary least squares (OLS) linear regression, all models were assessed for violation of the fundamental assumptions. There was no meaningful heteroskedasticity or autocorrelation in any models. While some multicollinearity was present in all models, for all models all variables were retained due to *a priori *theoretical considerations, or because of potential confounding effects identified during univariate analysis.

## Results

In total, 128 children < 19 years of age at the time of enrollment participated in the community-based health screenings. Almost ¾ (73%) of the children were from a family where at least one parent had completed more than a high school education, and almost ½ (47%) were covered by employer-sponsored dental insurance. While there was no difference between girls and boys with regard to dental insurance (*p *(Χ^2^) > 0.10), boys were more likely than girls to live in a household without a parent having completed more than high school (84% vs. 62%, respectively, *p *(Χ^2^) = 0.006). Other characteristics of participants included: average BMI percentile of 68.2 ± 31.8 (standard deviation) with 56% above the 85^th ^percentile (overweight or obese); average total cholesterol of 156.2 ± 27.7 mg/dL with 8% above 200 mg/dL; average HDL cholesterol of 41.7 ± 11.4 mg/dL with 48% below 40 mg/dL; average triglycerides of 72.6 ± 47.5 mg/dL with 21% above 150 mg/dL; and average fasting glucose of 89.5 ± 7.9 mg/dL with 7 participants having blood glucose 100-126 mg/dL.

Of the health screening participants, 91 children > 10 years of age, 118 parents (92% response rate for parents), and 90 parent-child pairs (where the child was > 10 years of age) completed the dental health survey. Summary descriptions of dental hygiene, attitudes, preventive practices, and self- or parent-assessed dental health are presented in Table 1 and the comparison of responses for parent-child pairs are presented and compared using a Χ^2 ^statistic in Table 2. Because Χ^2 ^analysis assesses for an association between variables not explainable by chance, in this case the presence of a statistically significant Χ^2 ^is interpreted to mean that responses between parent and child were not different. Results for the comparisons presented in Table 2 suggest that responses for parent-child dyads were very similar. Therefore, subsequent analyses (ANOVA and regression) present results using only parent survey responses to permit a larger sample size (particularly children < 10 years of age, who did not complete a survey).

**Table 1:** Self-Reported Dental Hygiene, Attitudes, Preventive Care, and Self-Assessed Dental Health as Reported by Participants (> 10 Years of Age Only) and Parents of Participants (Participants of All Ages), Unmatched

			Child	Parent
**Dental Hygiene Habits**	**Frequency of Brushing**	At Least Daily	91%	89%
		Less Than Daily	9%	11%
	**Frequency of Flossing**	At Least 2-6 Times Weekly	39%	47%
		Weekly or Less	61%	53%
**Dental Health Attitudes**	**Importance of Dental Health**	Very Important	59%	--
		Not Very - Somewhat	41%	--
	**Fear of Going to the Dentist**	Not at all Afraid	71%	--
		Some - Much Fear	29%	--
**Dental Health Preventive Care**	**Dental Health Care Home**	Has Regular Dental Home	94%	96%
		No Regular Dental Home	6%	4%
	**Last Dental Care Visit**	Within Last 6 Months	83%	84%
		More than 6 Months Ago	17%	16%
**Self- or Parent-Assessed Dental Health**	**Now or Ever Had a Cavity**	Yes	69%	61%
		No	31%	39%
	**Now or Ever Had a Filling**	Yes	63%	56%
		No/Don't Know	37%	44%
	**Now or Ever Had a Tooth Pulled**	Yes	48%	36%
		No/Don't Know	52%	64%
	**Overall Dental Health**	Excellent or Very Good	49%	55%
		Poor - Good	51%	45%

**Table 2:** Comparison of Self-Reported Dental Hygiene, Attitudes, Preventive Care, and Self-Assessed Dental Health as Reported by Participants (> 10 Years of Age Only) and Matched Responses by Their Parents

			Child	Parent	***p *(Χ**^**2**^**)**
**Dental Hygiene Habits**	**Frequency of Brushing**	At Least Daily	91%	87%	0.001
		Less Than Daily	9%	13%	
	**Frequency of Flossing**	At Least 2-6 Times Weekly	40%	51%	< 0.0001
		Weekly or Less	60%	49%	
**Dental Health Preventive Care**	**Dental Health Care Home**	Has Regular Dental Home	94%	97%	0.034
		No Regular Dental Home	6%	3%	
	**Last Dental Care Visit**	Within Last 6 Months	84%	86%	< 0.0001
		More than 6 Months Ago	16%	14%	
**Self- or Parent-Assessed Dental Health**	**Overall Dental Health**	Excellent or Very Good	49%	55%	< 0.0001
		Poor - Good	51%	45%	

Per parent report, the vast majority of children brushed their teeth daily (> 85%), though less than half flossed their teeth more than weekly. Also, most children in this sample reported having a dental home (> 90%) and a dental visit within the last 6 months (> 80%). More than ½ had a filling and/or cavity and more than ⅓ reported having had a tooth pulled. Approximately ⅔ of children reported that their dental health was very important, and almost ¾ said that they were not at all afraid of going to the dentist. Parents and children were similar in the overall assessment of their dental health: 49% of children and 55% of parents assessed overall dental health as excellent or very good (unmatched).

The univariate (ANOVA) associations between parent-reported dental hygiene, preventive dental care, and parent-assessed dental health and obesity are reported in Table 3, and the associations between parent-reported dental hygiene, preventive dental care, and parent-assessed overall dental health and markers of systemic inflammation are reported in Table 4. There was no statistically significant, univariate difference in the mean BMI z-score between any of the parent-reported variables for dental hygiene, preventive dental care, or parent-assessed overall dental health. There were, however, multiple univariate differences in markers of systemic inflammation and parent-reported variables for dental hygiene, preventive dental care, and parent-assessed overall dental health. In particular, dental hygiene (frequency of flossing) was significantly associated with glucagon-like protein (GLP)-1 and resistin. Frequency of preventive dental care was significantly associated with interferon (IFN)-γ, interleukin (IL)-10, myeloperoxidase (MPO), and tumor necrosis factor (TNF)-α. Finally, parent-assessed overall dental health was significantly associated with e-Selectin, haptoglobin, IL-1α, IL-6, IL-8, macrophage inflammation protein (MIP)-1α, soluble vascular cell adhesion molecule (sVCAM)-1, tissue plasminogen activator inhibitor (tPAI)-1, and vascular endothelial growth factor (VEGF).

**Table 3:** Univariate (ANOVA) Associations Between Parent-Reported Dental Hygiene, Preventive Dental Care, Self-Assessed Dental Health, and Obesity

		Obesity (Mean ± SD)		
**Dental Hygiene Habits**	**Flossing** (2-6 Times Weekly vs. Weekly or Less)	BMI z-Score	0.37 ± 1.2 vs. 0.64 ± 1.2	*p *> 0.1
**Dental Health Preventive Care**	**Preventive Care** (Every 6 Months vs. Annually or Less)	BMI z-Score	0.49 ± 1.2 vs. 0.81 ± 1.3	*p *> 0.1
**Parent-Assessed Dental Health**	**Overall Dental Health** (Excellent or Very Good vs. Poor - Good)	BMI z-Score	0.57 ± 1.1 vs. 0.54 ± 1.3	*p *> 0.1

**Table 4:** Univariate (ANOVA) Associations Between Parent-Reported Dental Hygiene, Preventive Dental Care, Parent-Assessed Dental Health, and Markers of Systemic Inflammation

		Marker of Systemic Inflammation* (Mean ± SD)		
**Dental Hygiene Habits**	**Flossing** (2-6 Times Weekly vs. Weekly or Less)	GLP-1 (pg/mL)	13.1 ± 7.3 vs. 22.1 ± 25.8	*p *= 0.079
		Resistin (pg/mL)	2.6e4 ± 9.4e4 vs. 3.6e4 ± 1.7e4	*p *= 0.043
**Dental Health Preventive Care**	**Preventive Care** (Every 6 Months vs. Annually or Less)	IFN-γ (pg/mL)	25.6 ± 78.7 vs. 111.5 ± 269.6	*p *= 0.062
		IL-10 (pg/mL)	10.4 ± 10.0 vs. 22.1 ± 40.6	*p *= 0.079
		MPO (pg/mL)	8.9e3 ± 4.4e3 vs. 1.8e4 ± 2.4e4	*p *= 0.013
		TNF-α (pg/mL)	3.6 ± 1.9 vs.8.4 ± 13.2	*p *= 0.013
**Parent-Assessed Dental Health**	**Overall Health** (Excellent or Very Good vs. Poor - Good)	e-Selectin (pg/mL)	38.9 ± 13.8 vs. 48.3 ± 18.6	*p *= 0.021
		Haptoglobin (ng/mL)	1.4e6 ± 8.0e5 vs. 9.0e5 ± 5.0e5	*p *= 0.004
		IL-1α (pg/mL)	983.1 ± 1169.1 vs. 514.6 ± 596.8	*p *= 0.057
		IL-6 (pg/mL)	31.7 ± 37.4 vs. 17.1 ± 23.9	*p *= 0.061
		IL-8 (pg/mL)	11.7 ± 12.2 vs. 6.5 ± 5.2	*p *= 0.029
		MIP-1α (pg/mL)	86.4 ± 79.3 vs. 50.1 ± 47.6	*p *= 0.038
		sVCAM-1 (pg/mL)	1674.5 ± 423.7 vs. 1914.9 ± 463.9	*p *= 0.028
		tPAI-1 (pg/mL)	10125.3 ± 5469.7 vs. 14741.6 ± 8404.4	*p *= 0.008
		VEGF (pg./mL)	167.8 ± 138.0 vs. 98.7 ± 134.9	*p *= 0.094

*Abbreviations for markers of systemic inflammation: GLP-1 (glucagon-like peptide-1); IFN-γ (interferon-gamma); IL (interleukin); MPO (myeloperoxidase); TNF-α (tumor necrosis factor-alpha); MIP-1α (macrophage inflammation protein-1-alpha); sVCAM-1 (soluble vascular cell adhesion molecule-1); tPAI-1 (tissue plasminogen activator inhibitor-1); VEGF (vascular endothelial growth factor)

The robustness of these univariate associations was assessed using linear OLS regression; the results from the statistically significant models are shown in Table 5. All models considered simultaneously a constant, age, gender, parent education, BMI z-score and one parent-reported variable for dental hygiene, preventive dental care, or parent-assessed overall dental health to predict a marker of systemic inflammation. Of the univariate associations reported in Table 4, the associations that persisted with OLS regression were those between parent-assessed overall dental health and e-Selectin, haptoglobin, sVCAM-1, and tPAI-1. Parent-assessed overall dental health was inversely associated with e-Selectin, sVCAM-1, and tPAI-1 and positively associated with haptoglobin. For all models, these associations were independent of associations between obesity and systemic inflammation, which was also statistically significant for all models.

**Table 5:** Results from Multiple Regression Analysis: Parent-Assessed Overall Dental Health, Obesity, and Systemic Inflammation

Dependent Variable*,**	n	**R**^**2**^	**Adjusted R**^**2**^	F Statistic	***p***_**F**_	Independent Variables	β***	**Standard Error**_**β**_	***p***_**β**_
e-Selectin	65	0.2	0.2	4.2	0.005	BMI z-Score	3.2	1.6	0.05
						Parent-Assessed Overall Dental Health	-10.1	3.9	0.01
Haptoglobin	69	0.2	0.2	5.5	0.001	BMI z-Score	1.6e5	6.2e4	0.01
						Parent-Assessed Overall Dental Health	4.1e5	1.5e5	0.008
sVCAM-1	67	0.4	0.2	3.5	0.01	BMI z-Score	-99.0	44.3	0.03
						Parent-Assessed Overall Dental Health	-218.0	106.6	0.04
tPAI-1	68	0.5	0.3	6.6	< 0.0001	BMI z-Score	2583.9	646.1	< 0.0001
						Parent-Assessed Overall Dental Health	-4927.8	1561.30	0.002

*All regression models included age, gender, and parent education in addition to BMI z-score, and parent-assessed overall dental health (coded as 0 = Overall Rating < Very Good (Poor-Good) and 1 = Overall Rating Excellent or Very Good)

**Abbreviations for markers of systemic inflammation: sVCAM-1 (soluble vascular cell adhesion molecule-1); tPAI-1 (tissue plasminogen activator inhibitor-1)

***β coefficients not standardized

## Discussion and Conclusions

This study assessed the association between parent-reported measures of dental hygiene, preventive care, and parent-assessed overall dental health with obesity and markers of systemic inflammation in children living in rural communities. This is among the first known studies to demonstrate a link between these self-reported measures of dental health and systemic inflammation independent of obesity in children. In this study, univariate analysis demonstrated statistically significant associations between parent-reported indicators of dental hygiene, preventive dental care, and parent-assessed overall dental health and multiple markers of systemic inflammation but not obesity. In OLS regression analysis, after controlling for age, gender, and socioeconomic status, parent-assessed overall dental health maintained statistically significant, *ceteris paribus *predictive effects on markers of systemic inflammation independent of obesity.

Results from this study, particularly that obesity was not univariately associated with indicators of dental hygiene, preventive care, or health, are consistent with previous reports of equivocal associations between dental caries and obesity [39]. Of note, however, was the finding that obesity and parent-assessed overall dental health were simultaneously and independently predictive of multiple markers of systemic inflammation. While the magnitude of relationships in OLS regression were modest, these relationships are consistent with a prevailing theory explaining the biologic mechanism linking oral health and obesity, and oral health and cardiovascular disease in adults. Relationships of smaller magnitude are expected in younger age groups, where pathogenic effects have not had sufficient time to accumulate and result in clinically detectable disease. Results from the current study provide preliminary evidence that poorer dental hygiene and oral health, which is presumed to lead to periodontal disease, and obesity may share a common, physiologic pathway in systemic inflammation. Further, longitudinal study is particularly recommended to understand the temporal development of these pathologies and how one may compound or contribute to the other.

It is important to acknowledge the limitations of this study. First, it is a cross-sectional, convenience sample of children living in rural, Appalachian communities and so the generalizability of results is unclear. Additionally and importantly, periodontal health was not directly measured, though self-reported dental health and hygiene behavior have been shown to be adequate proxies in epidemiologic or population-based studies [43]. Nevertheless, the results reported in this study using these epidemiologic and self-report survey methods would benefit from further study using more robust and direct measurements, including direct assessment of periodontal health using an oral exam, to ascertain whether directly assessed periodontal health is associated with local tissue inflammation as well as systemic inflammation. Additionally and consistent with requirements for establishing etiologic causality, determination of temporal associations is needed. Finally, both biochemical and statistical protocols used in this study contributed to cautious interpretation of results. The conservative criteria established for inclusion of inflammatory markers in the final analysis limited the sample size and, consequently, the regression analyses may be underpowered. However, this reduced sample sized combined with some multicollinearity both work to bias conclusions to accepting the null hypothesis of no effect and thus the observed effects of dental health habits on systemic inflammation are less likely to be attributable to type 1 error (false positive).

Results reported here will clearly benefit from additional studies in larger and more diverse populations. However, the initial results reported here suggest that poorer dental health in children may contribute to systemic pathophysiologic mechanisms common to obesity and cardiovascular disease. These observations, if affirmed by future, larger and more rigorous studies that include more robust measures of socioeconomic status and confounding by factors such as nutritional status and habits or other metabolic risk factors such as serum lipids or the metabolic syndrome, further underscore the importance of proactive dental health care as an integral component of holistic, overall health. Additionally, the findings of this study are consistent with recent interest in and calls for increased collaboration between primary care providers - both clinical (pediatricians) and dental (dentist and/or dental hygienist) - in identifying and addressing coincident obesity and poor dental health [44-47].

## Competing interests

The authors declare that they have no competing interests.

## Authors' contributions

SJF made substantial contributions to the conception and design of this study, participated in data collection and biochemical analysis, performed statistical analysis and interpretation of results, drafted and revised the final manuscript, and read and approved the final manuscript. CBC participated in data collection and biochemical analysis, participated in the interpretation of results, assisted in reviewing and revising the final manuscript, and read and approved the final manuscript. JCF made substantial contributions to the conception and design of this study, contributed to the data collection and biochemical analysis, participated in the interpretation of results, assisted in reviewing and revising the final manuscript, and read and approved the final manuscript. AGG contributed substantially to the data collection and biochemical analysis, participated in the interpretation of results, assisted in reviewing and revising the final manuscript, and read and approved the final manuscript. RJC made substantial contributions to the conception and design of this study, contributed to the data collection and biochemical analysis, participated in the interpretation of results, assisted in reviewing and revising the final manuscript, and read and approved the final manuscript.

## Pre-publication history

The pre-publication history for this paper can be accessed here:

http://www.biomedcentral.com/1472-6831/10/21/prepub
